# Myeloid A20 Regulates Periodontal Tissue Integrity and Temporomandibular Joint Structure

**DOI:** 10.1111/jre.70119

**Published:** 2026-05-20

**Authors:** Karol Alí Apaza Alccayhuaman, Patrick Heimel, Toni Dobsak, Stefan Tangl, Geert van Loo, Dieter D. Bosshardt, Reinhard Gruber

**Affiliations:** ^1^ Department of Oral Biology, University Clinic of Dentistry Medical University of Vienna Vienna Austria; ^2^ Core Facility Hard Tissue and Biomaterial Research, Karl Donath Laboratory, University Clinic of Dentistry Medical University of Vienna Vienna Austria; ^3^ Biomaterials and Technology, Department of Research University Center for Dental Medicine Basel UZB, University of Basel Basel Switzerland; ^4^ Ludwig Boltzmann Institute for Experimental and Clinical Traumatology in the AUVA Trauma Research Center Austrian BioImaging/CMI Vienna Austria; ^5^ Austrian Cluster for Tissue Regeneration Vienna Austria; ^6^ VIB Inflammation Research Center Ghent University Gent Belgium; ^7^ Department of Biomedical Molecular Biology Ghent University Gent Belgium; ^8^ Department of Periodontology, School of Dental Medicine University of Bern Bern Switzerland

**Keywords:** A20/TNFAIP3, alveolar bone, NF‐κB, osteoimmunology, periodontium, temporomandibular joint

## Abstract

**Aim:**

To determine whether myeloid‐specific deletion of A20 (TNFAIP3), a key negative regulator of NF‐κB signaling, affects periodontal supporting tissues and temporomandibular joint integrity under baseline conditions.

**Methods:**

A20^*myel‐KO*^ and wild‐type littermates were analyzed at 10–11 weeks of age using high‐resolution micro–computed tomography and histology. Alveolar bone architecture, periodontal ligament space, epithelial morphology, and temporomandibular joint (TMJ) compartments were evaluated through quantitative micro‐CT measurements and qualitative histological assessment.

**Results:**

A20^myel‐KO^ mice exhibited a craniofacial structural phenotype characterized by reduced alveolar bone volume fraction, widening of the periodontal ligament space, and altered dentoalveolar morphology. Histological analysis revealed epithelial architectural changes. In the TMJ, micro‐CT demonstrated reduced bone volume fraction, accompanied by histological evidence of trabecular bone loss and altered condylar growth plate organization.

**Conclusion:**

A20‐mediated regulation in myeloid cells contributes to the maintenance of periodontal and craniofacial skeletal homeostasis. Loss of this regulatory pathway was associated with structural alterations involving alveolar bone loss, periodontal ligament architecture, and temporomandibular joint morphology, suggesting that dysregulated myeloid inflammatory signaling may influence multiple craniofacial skeletal compartments even in the absence of experimentally induced inflammation.

## Clinical Relevance Box

**Background:**

- Periodontal tissues are continuously exposed to microbial and mechanical stimuli, requiring tight regulation of myeloid inflammatory signaling to maintain bone and soft tissue homeostasis.
- A20 (TNFAIP3) is a key negative regulator of NF‐κB signaling, but its role in periodontal and craniofacial tissue maintenance under baseline conditions remains unclear.

**Added value of this study:**

- Myeloid‐specific A20 deficiency was associated with alveolar bone loss, widening of the periodontal ligament space, epithelial alterations, and structural deterioration of the temporomandibular joint in the absence of induced inflammation.
- These findings identify myeloid A20 as an essential regulator of osteoimmune balance across periodontal and craniofacial skeletal sites.

**Clinical implications:**

- Impaired intrinsic control of myeloid inflammatory signaling may contribute to individual susceptibility to periodontal tissue breakdown.
- Regulatory pathways such as the A20–NF‐κB axis may represent potential targets for limiting inflammatory bone loss while preserving host defense.

## Graphical abstract

Dysregulation of A20 signaling has been implicated in several immune‐mediated diseases, including rheumatoid arthritis and psoriasis, conditions characterized by excessive NF‐κB activation and inflammatory tissue damage. The structural alterations observed in this model suggest that impaired regulation of innate immune signaling may increase susceptibility to inflammatory bone loss and reduce tissue repair capacity. Although periodontitis was not experimentally induced in this study, these findings support the concept that host regulatory pathways, such as the A20–NF‐κB axis, may influence individual susceptibility to periodontal and craniofacial tissue breakdown.

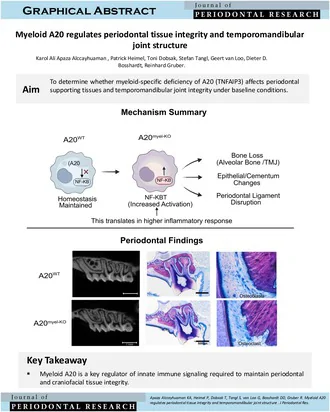

## 1 Introduction

The periodontium provides structural support and functional anchorage for teeth and consists of four integrated tissues: gingiva, alveolar bone, periodontal ligament, and cementum [1]. Beyond its mechanical role, the periodontium functions as a dynamic immunological barrier that maintains homeostasis at the interface between host tissues and the oral microbiota [2, 3]. A key component of this barrier is the junctional epithelium, which forms a semi‐permeable seal rather than an absolute physical blockade, permitting controlled translocation of microbial‐derived ligands into the underlying connective tissue and alveolar bone marrow [4]. This physiological exposure continuously activates innate immune cells of the myeloid lineage, including neutrophils, monocytes, and macrophages, through pattern‐recognition receptors and antigen‐presentation pathways [4].

Basal activation of myeloid cells is now recognized as an integral component of periodontal tissue homeostasis. Evidence from germ‐free mouse models demonstrates reduced alveolar bone loss in the absence of microbial stimulation, indicating that physiological bone resorption is a normal consequence of immune surveillance within the periodontium [4]. Under pathological conditions, however, persistent microbial challenge and dysregulated immune responses shift this balance toward excessive bone resorption, ultimately resulting in periodontitis [5]. Neutrophils and macrophages play central roles in orchestrating inflammatory responses and microbial clearance [6], while osteoclasts act as the terminal effectors of bone resorption [6]. Given the constant immune stimulation imposed by the oral microbiota, precise molecular mechanisms must exist to restrain inflammatory signaling and prevent excessive tissue destruction. Importantly, both the dentoalveolar complex and the temporomandibular joint undergo continuous immune‐regulated remodeling within the craniofacial skeleton. Consequently, dysregulation of myeloid inflammatory signaling may influence both compartments. Examining these structures within the same experimental framework therefore provides insight into whether altered immune regulation produces a broader craniofacial skeletal phenotype.

The temporomandibular joint (TMJ), a complex synovial articulation, is composed of the mandibular condyle, articular disc, and temporal glenoid fossa [7]. TMJ integrity depends on continuous remodeling of cartilage and bone to accommodate mechanical loading during mastication. Disruption of this tightly regulated process can result in inflammatory joint degeneration, pain, and functional impairment [7]. The TMJ is frequently affected in systemic inflammatory diseases, including rheumatoid arthritis and juvenile idiopathic arthritis [8], underscoring its susceptibility to immune‐mediated tissue remodeling. Experimental models of chronic inflammation, such as tumor necrosis factor‐α (TNF‐α) transgenic mice, develop severe TMJ pathology, highlighting the importance of inflammatory signaling pathways in joint homeostasis [9, 10]. However, the contribution of intrinsic immune regulatory mechanisms within myeloid cells to TMJ integrity remains incompletely understood.

A20, encoded by TNFAIP3, is a ubiquitin‐editing enzyme that functions as a critical negative regulator of NF‐κB signaling, thereby limiting the magnitude and duration of inflammatory responses. Genetic or functional impairment of A20 is associated with exaggerated inflammatory responses and has been implicated in multiple immune‐mediated diseases, including periodontitis [11]. In gingival tissues, A20 expression has been reported to differ between healthy and inflamed conditions [12, 13], and experimental studies have shown that A20 regulates cytokine production in macrophages and epithelial cells and modulates inflammatory responses in oral tissues [14]. In ligature‐induced periodontitis, heterozygous A20 knockout promoted the inflammatory changes and bone loss [15]. Consistently, A20 depletion in macrophages and gingival keratinocytes enhanced cytokine secretion [16]. Beyond the oral cavity, myeloid‐specific A20 deficiency induces erosive polyarthritis [17] and critically affects macrophage polarization and mucosal immunity [18, 19]. Moreover, osteoclast‐specific A20 deficiency promotes systemic bone loss [20]. Collectively, these findings support a role for A20 in regulating inflammatory bone remodeling and raise the question of whether myeloid‐specific A20 deficiency also affects craniofacial tissues under baseline conditions.

Because both the dentoalveolar complex and the temporomandibular joint belong to the craniofacial skeleton and undergo continuous remodeling influenced by immune‐regulated turnover, dysregulation of myeloid inflammatory signaling may affect both compartments. The aim of the present study was therefore to determine the impact of myeloid‐specific A20 deficiency on craniofacial bone homeostasis under baseline conditions, with a particular focus on the dentoalveolar complex and the temporomandibular joint. We hypothesized that loss of A20‐mediated regulation in myeloid cells may alter immune‐bone interactions within the craniofacial skeleton, resulting in detectable structural changes in periodontal tissues and the TMJ even in the absence of experimentally induced inflammation. To test this hypothesis, we used high‐resolution micro–computed tomography and histological analyses to assess alveolar bone architecture, periodontal ligament morphology, and TMJ integrity in myeloid‐specific A20‐deficient mice.

## 2 Methods

### 2.1 Mice and μCT Analysis

Male and female A20^FL/FL^; LysM‐Cre Tg/+ mice, which exhibit a myeloid‐specific knockout of A20 (A20^*myel‐KO*^ *)*, and their wild‐type littermates (A20^FL/FL^; LysM‐Cre +/+) were those reported and provided by Geert van Loo (VIB Inflammation Research Center, Ghent University, Ghent, Belgium) [17]. The study included eleven animals in total (six males and five females). Mice were euthanized at 10–11 weeks of age, and their tissues were fixed in 4% phosphate‐buffered formalin (Roti‐Histofix, Carl Roth, Karlsruhe, Germany). The skulls of five wild‐type (A20^*WT*^) and six A20^*myel‐KO*^ mice were analyzed using high‐resolution micro‐computed tomography (μCT) with a SCANCO μCT 50 system (Scanco Medical AG, Brüttisellen, Switzerland). Scans were performed at a voxel size of 10.3 μm, using a voltage of 90 kV, a current of 114 μA, and an integration time of 300 ms. The analysis focused on the maxilla, where teeth, periodontal ligament (PDL) space, and alveolar bone were evaluated for morphological changes. Temporomandibular joint (TMJ) structures, including the mandibular condyle and temporal glenoid fossa, were also analyzed. TMJ structures were automatically segmented using Definiens Developer XD Version 2.1.1 software (Definiens, Munich, Germany). All image analyses were performed blinded to genotype. Specimens were assigned coded identifiers prior to analysis to ensure that investigators performing segmentation and measurements were unaware of the experimental group.

### 2.2 Periodontal Structures by μCT

Segmentation of bone and teeth was performed using Amira 6.1.1 software (Thermo Fisher Scientific, Waltham, MA, USA). B‐splines were placed along the cemento*–*enamel junction (CEJ) and alveolar bone crest (ABC) at buccal and palatal sites of the first to third molars (M1–M3). Distances from CEJ to ABC were measured every 50 μm via linear regression, with measurements taken perpendicular to the CEJ plane. Periodontal parameters were quantified using a custom Definiens rule set, including molar volume (MV), PDL space volume (mm^3^), bone volume (mm^3^), tissue volume (mm^3^), and the bone volume‐to‐tissue volume ratio (%) within the M1–M3 region of interest.

### 2.3 Temporomandibular Joint by μCT

For the TMJ, mandibular bone erosion (μm) and bone volume/tissue volume ratio (%) were analyzed for both the mandibular condyle and articular fossa. Erosion was quantified by calculating the average distance from a smoothed computational surface to the actual bone surface. A 200 μm zone from the smoothed surface was used to determine bone and tissue volumes. Surface tension‐constrained region growing was applied to smooth bone edges, with the condylar surface projected dorsally onto the articular fossa to define the region of interest.

### 2.4 Histological Processing and Analysis of Periodontal Structures and TMJ

After dehydration in alcohol, skulls were embedded in light‐cured resin (Technovit 7200 VLC + BPO; Heraeus Kulzer & Co., Wehrheim, Germany). Thin‐ground sections of A20^*myel‐KO*^ and A20^*WT*^ littermates were prepared using Exakt Cutting and Grinding equipment (Exact Apparatebau, Norderstedt, Germany). Coronal sections were obtained at the level of the TMJ and second molar (M2), and stained using the Levai‐Laczko technique [21]. Digital images were captured using an Olympus BX61VS microscope with the dotSlide 2.4 digital virtual microscopy system (Olympus, Tokyo, Japan) at 321.5 nm per pixel resolution. Histological evaluation was performed to qualitatively assess tissue morphology and to provide structural context for the alterations identified by micro‐CT. In addition, histology enabled evaluation of soft tissue structures, which cannot be resolved using micro‐computed tomography. Quantitative morphometric analyses were primarily performed using micro‐CT, which enables high‐resolution three‐dimensional measurements of bone and periodontal structures.

### 2.5 Statistical Analysis

Descriptive statistics were performed for all dentoalveolar and TMJ parameters obtained from micro‐CT. A linear mixed model (LMM) was applied to account for the nested structure of the data, where each subject contributed repeated measurements (M1, M2, and M3) at a single time point. Fixed effects in the model included group (A20^*myel‐KO*^ vs. A20^*WT*^), bone volume fraction (BV/TV), molar volume, and periodontal ligament space area (PLS.Ar), each analyzed per molar site. Subject ID was included as a random effect to control for intra‐individual correlations. Significance was set at *p* < 0.05. CEJ–ABC distances were evaluated using regression analysis to assess group‐related morphological changes. Histological findings were assessed qualitatively. Both left and right sides of the alveolar bone, teeth, and TMJ were analyzed independently, yielding 10 A20^*WT*^ and 12 A20^myel‐KO^ anatomical sites. Effect sizes were estimated using Cohen's d for pairwise comparisons and η^2^ (eta‐squared) for the LMM, quantifying the magnitude of between‐group differences. Bonferroni correction was applied to adjust for multiple testing in post hoc analyses.

## 3 Results

### 3.1 Dentoalveolar Complex: Overview of Analytical Approach

Given that myeloid‐specific A20 deficiency has been associated with systemic bone loss, we examined whether similar catabolic changes affect the dentoalveolar complex. Using a combination of high‐resolution μCT and histology, we assessed alveolar bone architecture, periodontal ligament (PDL) space, and cemento–alveolar relationships across molar sites. This multimodal approach enabled detection of osteoporotic features and site‐specific alterations within the periodontium. While gross three‐dimensional reconstructions appeared largely comparable between genotypes, cross‐sectional μCT images and corresponding histological sections revealed distinct internal structural differences in A20^*myel‐KO*^ mice, including expansion of the PDL space and altered trabecular architecture (Figure 1).

**FIGURE 1:**
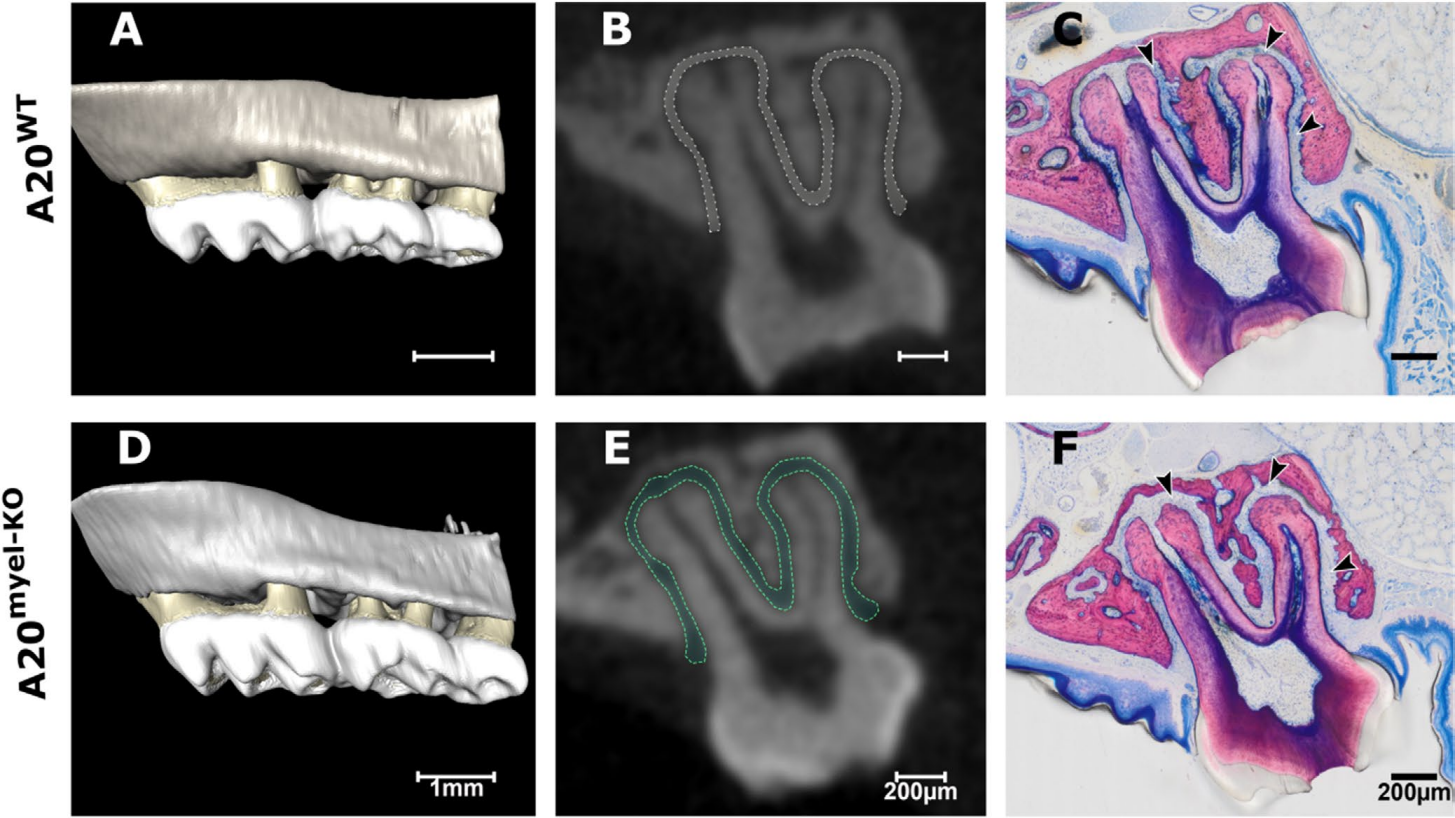
Dentoalveolar complex in A20^*WT*^ and A20^*myel‐KO*^ mice. Three‐dimensional micro‐CT reconstructions of A20^*WT*^ (A) and A20^*myel‐KO*^ (D) mice appear similar at first glance. However, cross‐sectional micro‐CT images (B, E) reveal distinct internal structural differences in A20^*myel‐KO*^ mice, which are corroborated by histological sections (C, F) obtained at the same anatomical level. The periodontal ligament (PDL) space surrounding the second molar (M2) is expanded in A20^*myel‐KO*^ mice, compared to the A20^*WT*^ controls. The PDL boundaries are indicated by dashed gray lines in A20^*WT*^ (B) and green lines for A20^*myel‐KO*^ (E). Arrows in the histological sections (C, F) highlight the widened PDL region.

### 3.2 Micro‐CT Analysis of Alveolar Bone, Tooth Volume, and Periodontal Ligament Space

Segmentation‐based quantitative analysis demonstrated clear differences in alveolar bone structure between groups. A20^*WT*^ mice exhibited a denser and more cortical‐like alveolar bone with fewer and larger marrow voids, whereas A20^*myel‐KO*^ mice showed a more porous, trabecular pattern across molar regions (Figure 2A,B). Consistent with these observations, bone volume fraction (BV/TV) was significantly reduced in A20^*myel‐KO*^ mice across all analyzed molar sites (Figure 2C). Tooth volume was also modestly but significantly reduced in A20^*myel‐KO*^ mice at the first (M1) and second molars (M2), while no difference was detected at the third molar (M3) (Figure 2D). In parallel, analysis of the periodontal ligament space revealed a significant widening in A20^*myel‐KO*^ mice, most pronounced around M2 (*p* = 0.009), whereas M3 was largely unaffected (Figure 2E). Together, these findings indicate that myeloid A20 deficiency compromises alveolar bone integrity and alters dentoalveolar spatial relationships in a site‐dependent manner.

**FIGURE 2:**
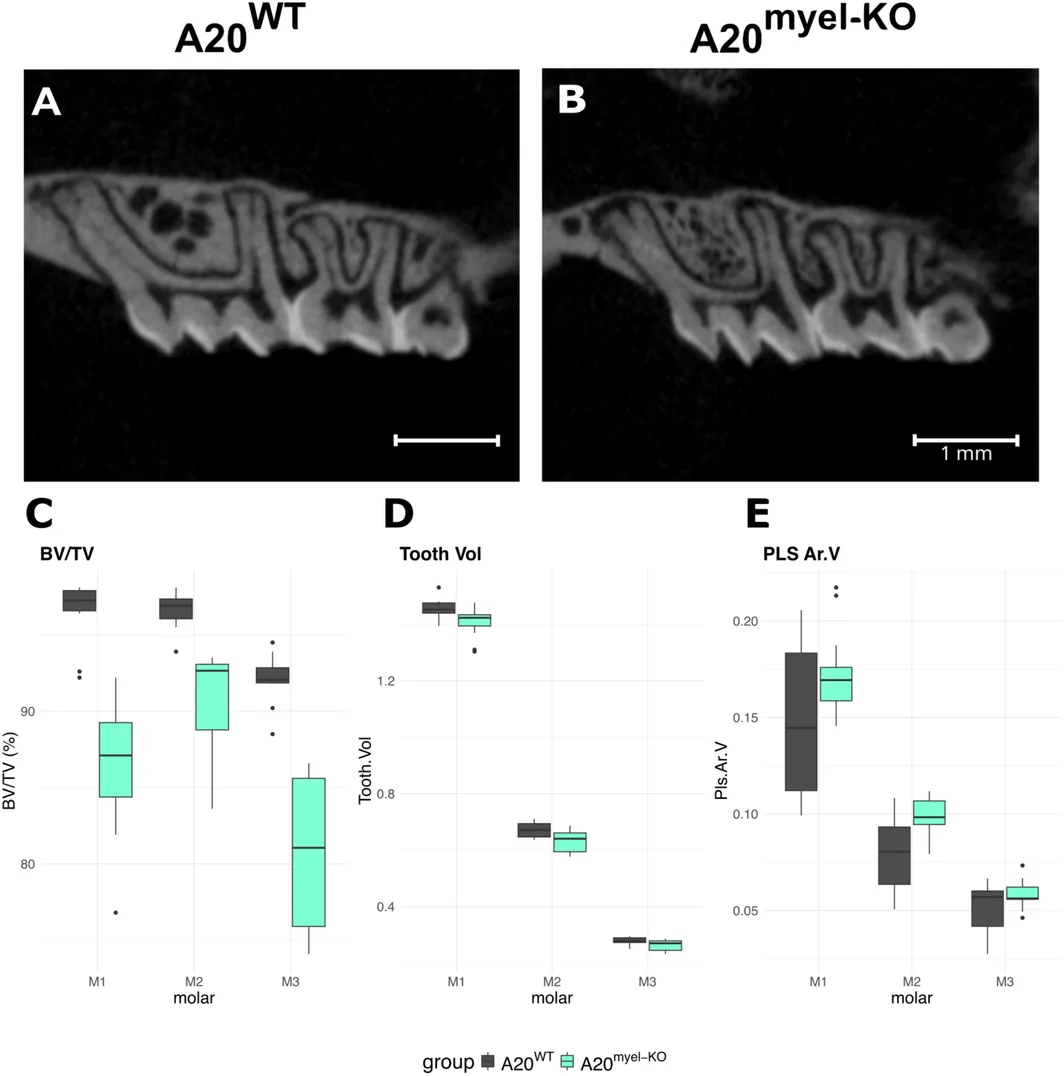
Micro‐CT analysis of local bone, tooth volume, and periodontal space. Representative sagittal micro‐CT sections of the alveolar complex show trabecular bone morphology in the A20^*WT*^ (A) and A20^*myel‐KO*^ (B) mice. Quantitative analysis of bone volume fraction (BV/TV) (C) demonstrates significantly higher values in A20^*WT*^ mice at the first (M1) and second (M2) molars compared with A20^*myel‐KO*^, whereas no significant differences were observed at the third molar (M3). Tooth volume analysis (D) shows significantly larger volumes in A20^*WT*^ mice at M1 and M2, whereas M3 volumes are comparable between groups. Analysis of periodontal ligament space area (PLS.Ar) (E) reveals widening of the PDL space in A20^*myel‐KO*^ mice relative to A20^*WT*^ controls.

### 3.3 Micro‐CT Assessment of Cemento–Alveolar Relationships

To further characterize vertical alveolar bone levels, distances between the cemento*–*enamel junction (CEJ) and alveolar bone crest (ABC) were quantified at buccal and palatal aspects of individual molars. On the buccal side, CEJ–ABC distances were significantly greater in A20^*WT*^ (Figure 3A) mice compared with A20^*myel‐KO*^ mice (Figure 3D) at M2 and M3 (Figure 3C). On the palatal side, a significant difference was observed at M1, with greater CEJ–ABC distances in A20^*WT*^ mice (Figure 3B), whereas no significant differences were detected at M2 and M3 (Figure 3F) compared to A20^*myel‐KO*^ mice (Figure 3E). These measurements describe geometric cemento–alveolar relationships rather than classical periodontal bone loss. Because A20^*myel‐KO*^ mice exhibited alterations in tooth volume and periodontal ligament space dimensions, the spatial relationship between the cemento*–*enamel junction and the alveolar bone crest differs from that observed in conventional periodontal bone loss measurements and should therefore be interpreted in the context of the overall dentoalveolar morphology.

**FIGURE 3:**
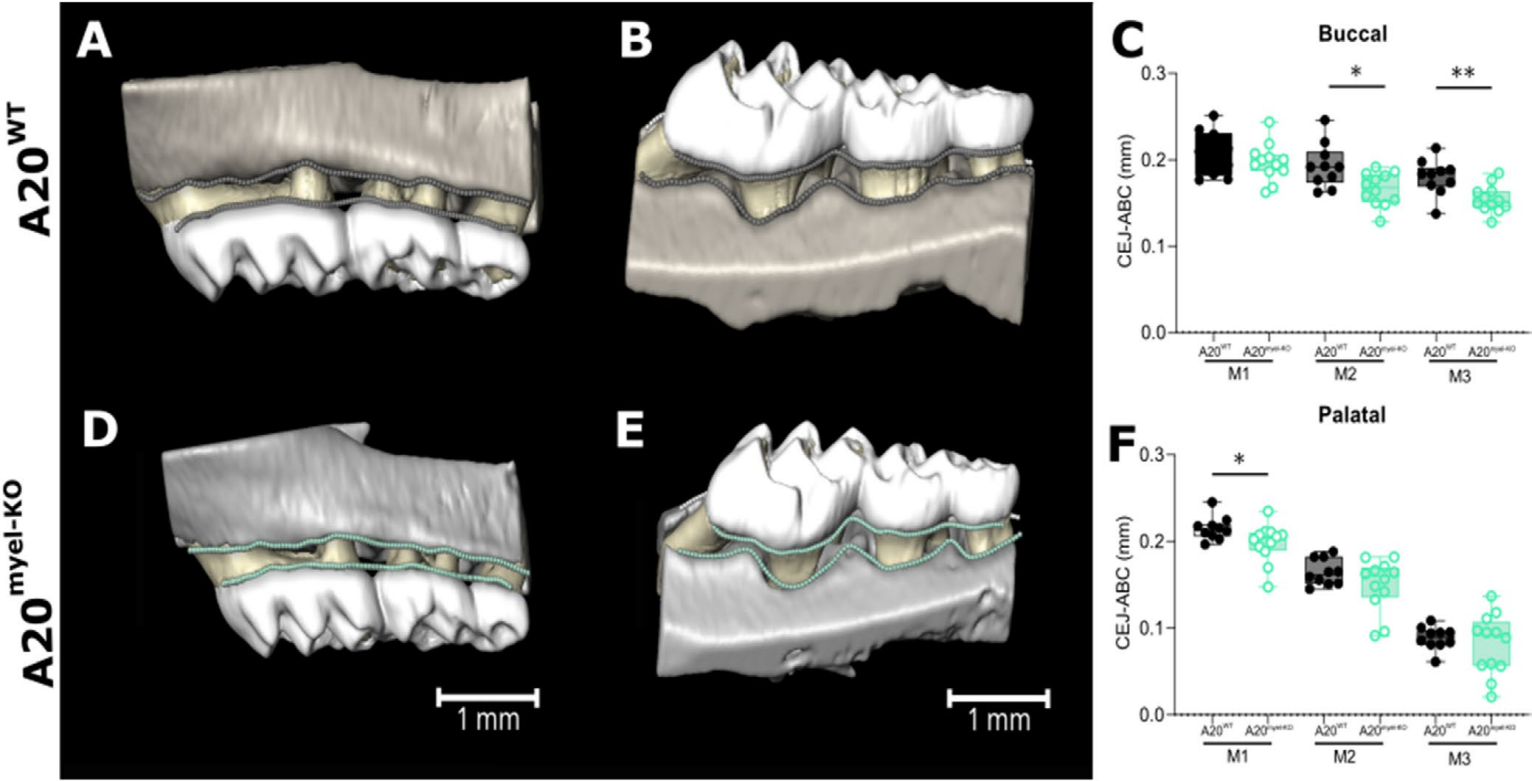
Volume rendering of A20^*WT*^ and A20^*myel‐KO*^ specimens with B‐spline landmark mapping. Three‐dimensional micro‐CT reconstructions of A20^*WT*^ (A, B) and A20^*myel‐KO*^ (D, E) specimens with B‐spline landmarks placed along the buccal (A, D) and palatal (B, E) molar surfaces to define the cemento–enamel junction (CEJ) and alveolar bone crest (ABC). The CEJ–ABC distance was calculated to assess vertical alveolar bone levels. Quantitative analysis of CEJ–ABC distances is shown for buccal (C) and palatal (F) aspects across individual molars. Significantly greater CEJ–ABC distances were observed in A20^*WT*^ mice compared with A20^*myel‐KO*^ mice at the buccal aspect of the second and third molars (M2 and M3) and at the palatal aspect of the first molar (M1).

### 3.4 Histological Evaluation of Mandibular Trabecular Bone

Histological analysis of mandibular sections corroborated the μCT findings. In A20^*WT*^ mice, trabecular bone appeared dense and well‐organized, with thick trabeculae and limited marrow spaces. In contrast, A20^*myel‐KO*^ mice exhibited thinner trabeculae, enlarged marrow cavities, and focal resorptive features, consistent with catabolic bone remodeling (Figure 4). These changes further support the presence of a low–bone‐mass phenotype affecting craniofacial skeletal sites in the absence of A20 in myeloid cells.

**FIGURE 4:**
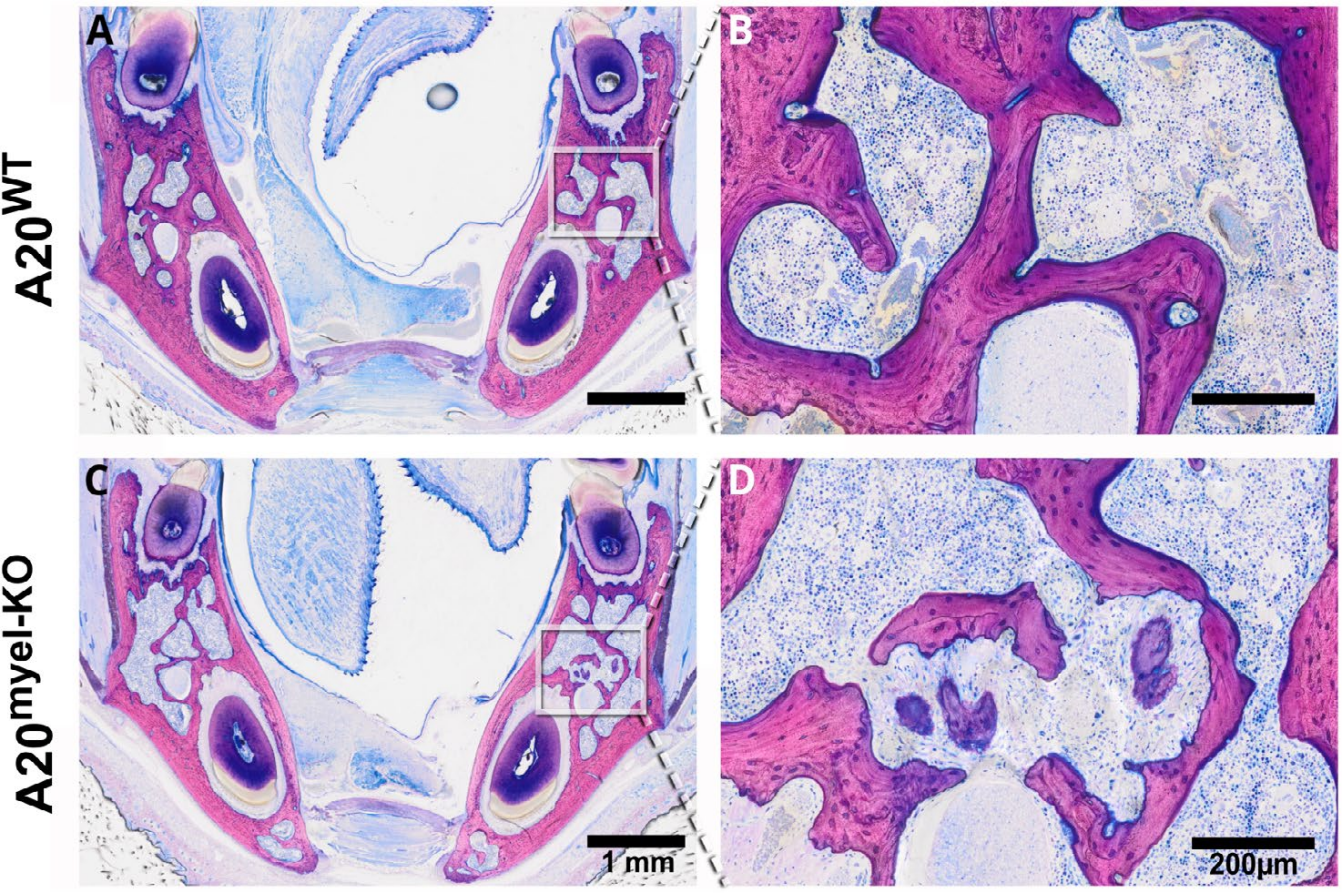
Representative histological sections of the dentoalveolar bone in A20^*WT*^ and A20^*myel‐KO*^ mice. Levai–Laczko–stained coronal sections of the mandible illustrating dentoalveolar bone morphology in A20^*WT*^ (A, B) and A20^*myel‐KO*^ (C, D) mice. Low‐magnification images (A, C) show the overall architecture of the dentoalveolar complex. Higher‐magnification views of the boxed regions (B, D) highlight differences in trabecular morphology. A20^*WT*^ specimens (B) display thicker and more compact trabeculae, whereas A20^*myel‐KO*^ specimens (D) show thinner and more irregular trabecular structures.

### 3.5 Histological Analysis of Alveolar Bone

To further characterize the craniofacial skeletal phenotype, we examined the maxillary alveolar bone architecture surrounding the molars (Figure 5). Structural differences were observed between genotypes. In A20^*myel‐KO*^ mice (Figures 5E–H), the buccal plate was noticeably thinner compared to A20^*WT*^ controls (Figures 5A–D), and multinucleated giant cells consistent with osteoclast‐like morphology were observed in the crestal region.

**FIGURE 5:**
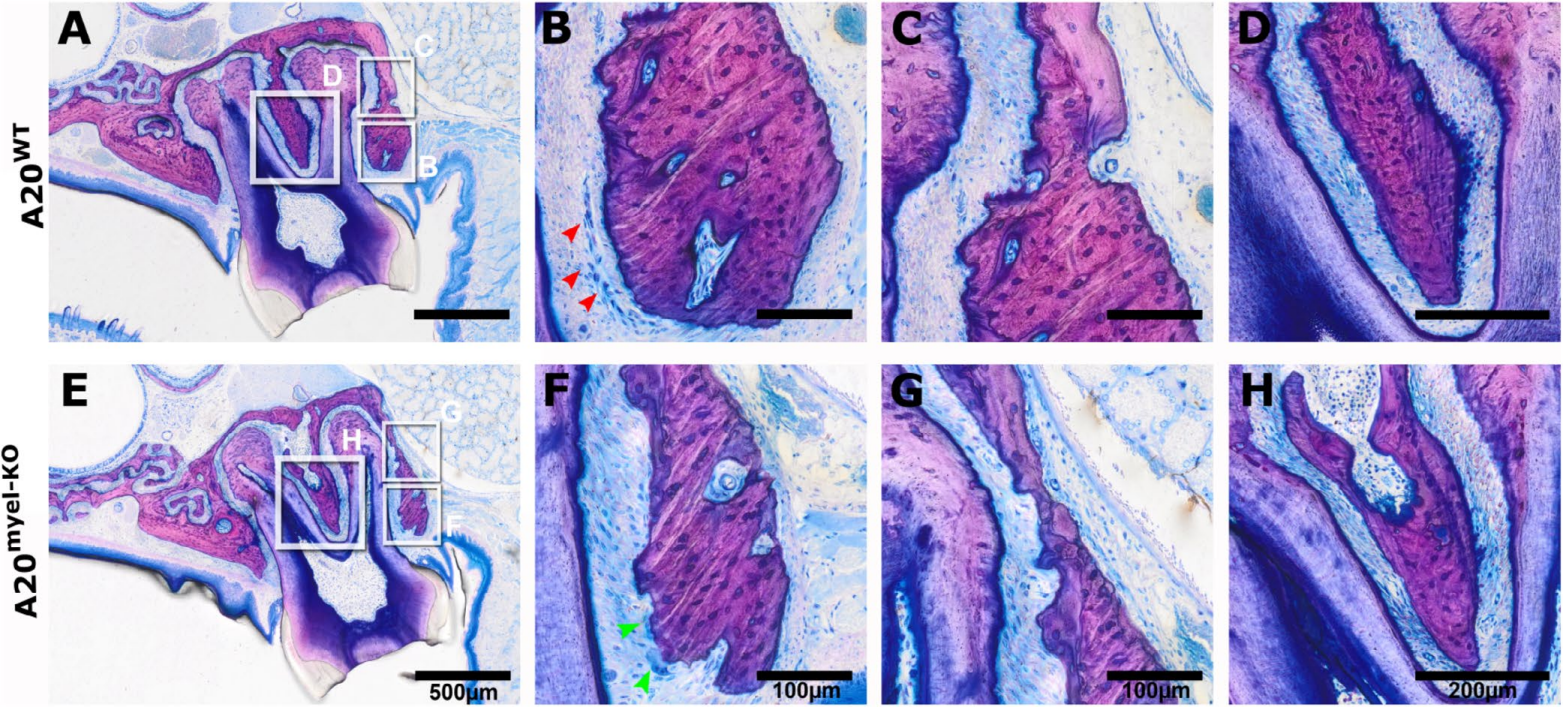
Histological evaluation of alveolar bone in A20^*WT*^ and A20^*myel‐KO*^ mice. Levai–Laczko–stained coronal sections of the maxillary dentoalveolar region. Low‐magnification images illustrate the overall architecture in A20^*WT*^ (A) and A20^*myel‐KO*^ (E) mice. The boxed regions correspond to the higher magnification views of the buccal alveolar crest (B‐F), mid buccal plate (C‐G), and interradicular bone (D, H). In A20^*WT*^specimens, the buccal alveolar crest (B) shows osteoblast‐like cells lining the bone surface (red arrow). In contrast, A20^*myel‐KO*^ (F) shows multinucleated cells consistent with osteoclast‐like morphology associated with bone resorption (green arrows). The mid‐buccal plate in A20^*myel‐KO*^ mice (G) exhibits focal narrowing compared with the corresponding region in A20^*WT*^ controls (C). The interradicular bone is also affected in A20^*myel‐KO*^ mice (H), showing localized resorptive areas compared with the more compact interradicular bone observed in A20^*WT*^ controls (D).

Structural alterations extended into the interradicular bone, where A20^*myel‐KO*^ mice exhibited enlarged marrow spaces and localized remodeling/resorptive changes compared with the denser interradicular architecture in A20^*WT*^ mice. Collectively, these histological findings are consistent with the μCT‐based evidence of reduced bone volume and highlight the vulnerability of alveolar bone to catabolic remodeling in the absence of myeloid A20 signaling.

### 3.6 Histological Analysis of Oral and Periodontal Soft Tissues

To evaluate whether periodontal soft tissues were affected in A20^*myel‐KO*^, we examined the oral lining mucosa, junctional epithelium, and palatal mucosa (Figure 6). In A20^*WT*^ mice, the oral mucosa displayed a well‐organized stratified squamous epithelium characterized by a polarized basal layer, a prominent stratum spinosum, and well‐defined epithelial projections into the lamina propria (Figure 6B). In contrast, A20^*myel‐KO*^ mice exhibited a flatter epithelial profile with reduced epithelial projections and thinning of the suprabasal layers, indicating attenuated epithelial differentiation (Figure 6F).

**FIGURE 6:**
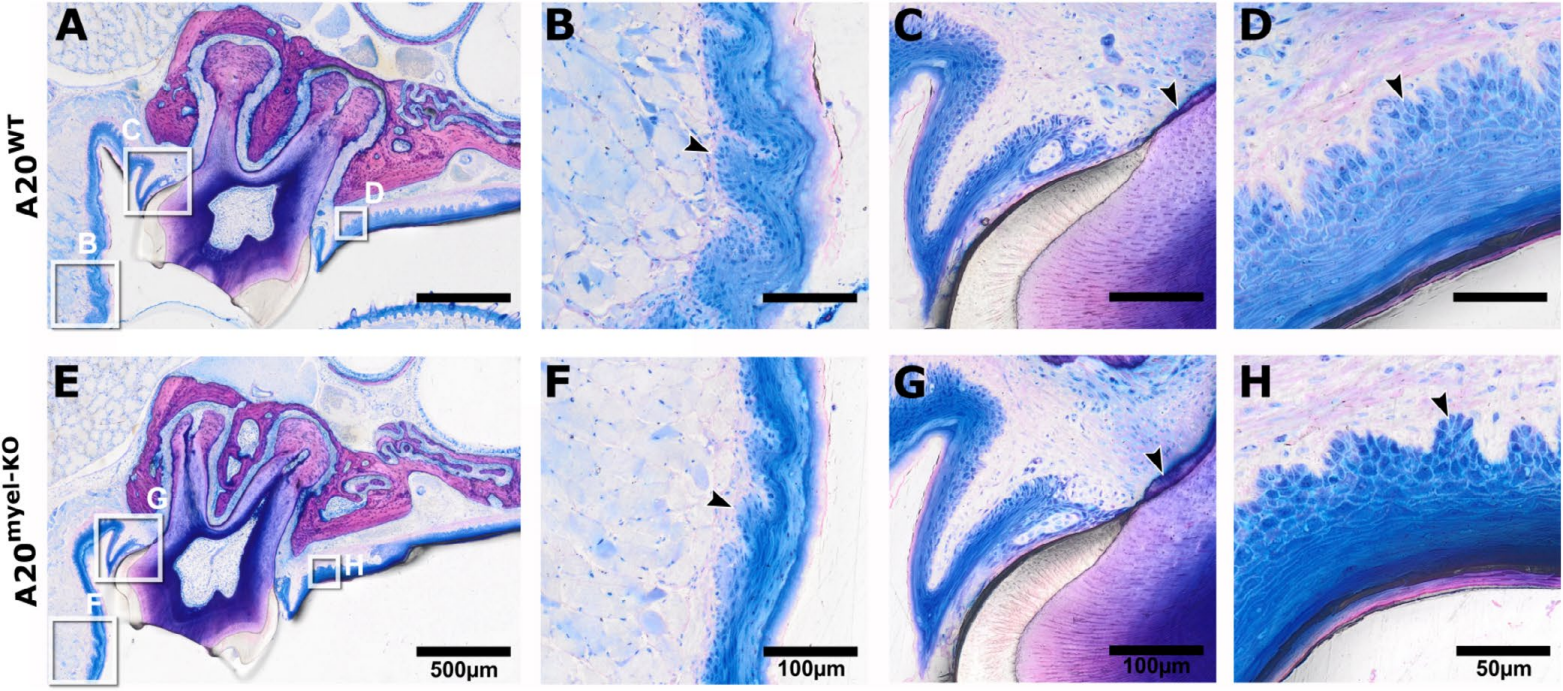
Impact of A20 deficiency on oral mucosal and periodontal soft tissue morphology. Levai–Laczko–stained coronal sections of the maxillary oral mucosa and gingival tissues. Low‐magnification images illustrate the overall architecture in A20^*WT*^ (A) and A20^*myel‐KO*^ mice (E). The boxed regions correspond to higher‐magnification views of the oral lining mucosa (B, F), the gingival region adjacent to the cemento–enamel junction (C, G), and the hard palate mucosa (D, H). In the oral lining mucosa, arrows indicate epithelial projections (rete ridges) extending into the lamina propria, which appear less pronounced in A20^*myel‐KO*^ specimens. In the gingival region adjacent to the cemento–enamel junction (CEJ), both groups maintain gingival architecture; however, A20^*myel‐KO*^ mice show thickening of the cementum layer at the CEJ (G, arrows). The hard palate mucosa in (D, H) displays an intact epithelial layer in both genotypes, with subtle qualitative differences in the appearance of the keratinized surface layer between groups.

Both genotypes preserved the typical non‐keratinized morphology of the junctional epithelium (Figure 6C,G); however, A20^*myel‐KO*^ mice consistently showed a markedly thickened cementum layer at the CEJ, whereas A20^*WT*^ mice maintained the expected thin, acellular cementum. In the hard palate (Figure 6D,H), the orthokeratinized epithelium was intact in both groups, but A20^*myel‐KO*^ specimens demonstrated a subtle qualitative difference in the stratum corneum compared to controls.

### 3.7 Temporomandibular Joint Phenotype by μCT

In the A20^*myel‐KO*^ model, micro‐CT analysis revealed compromised integrity of the condylar surface compared to the control mice (Figure 7A,B). To quantify these changes, we measured the bone volume fraction (BV/TV) at key TMJ regions, including the condylar head and temporal fossa. A20^*WT*^ mice exhibited significantly higher BV/TV values at the condylar head (96.6% vs. 89.3%; *p* < 0.0001) and the temporal fossa (95.2% vs. 85.2%; *p* < 0.0001) compared to A20^*myel‐KO*^ mice. Further analysis of the condylar surface confirmed additional deterioration in the A20^*myel‐KO*^ group (84.3% vs. 88.8%; *p* = 0.047). Three‐dimensional micro‐CT renderings visually illustrate surface erosion in A20^*myel‐KO*^ mice, corroborating the quantitative findings. Although gross morphological differences were not immediately evident, these microstructural alterations highlight functional impairment associated with myeloid‐specific A20 deficiency.

**FIGURE 7:**
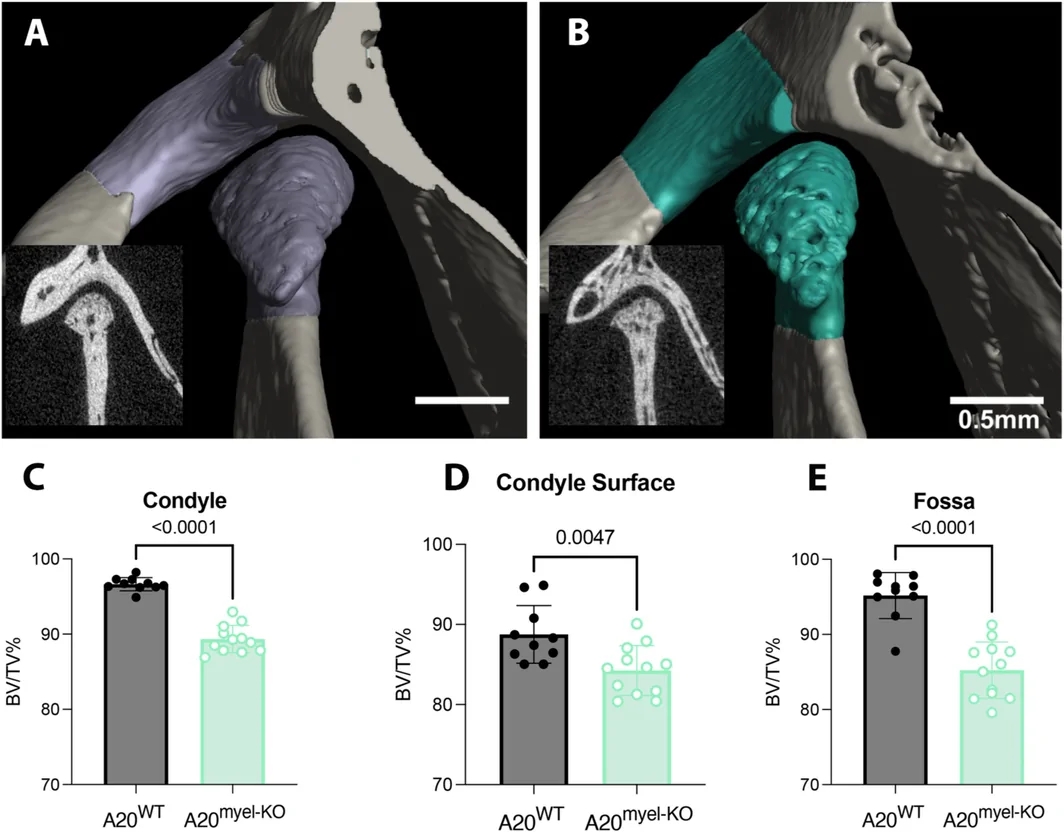
Quantitative μCT analysis of the temporomandibular joint (TMJ). Three‐dimensional micro‐CT reconstructions of the TMJ in A20^*WT*^ (A) and A20^*myel‐KO*^ (B) mice, illustrating the anatomical regions analyzed, including the mandibular condyle and temporal fossa. Insets show representative coronal micro‐CT slices of the corresponding TMJ region. Quantitative analysis of bone volume fraction (BV/TV) is shown for the condylar head (C), condylar surface (D), and temporal fossa (E). A20^*myel‐KO*^ mice exhibited significantly reduced BV/TV compared with A20^*WT*^ controls across all analyzed TMJ compartments.

### 3.8 Temporomandibular Joint Phenotype by Histology

Histological evaluation of the temporomandibular joint revealed structural differences between A20^*WT*^ and A20^*myel‐KO*^ mice (Figure 8). In A20^*WT*^ controls, the condylar cartilage displayed a normal and orderly progression from the proliferative and hypertrophic zones to the underlying ossified bone, consistent with preserved endochondral ossification. In contrast, A20^*myel‐KO*^ mice showed a substantially thickened proliferative zone and a disorganized hypertrophic layer, indicative of impaired chondrocyte maturation and disrupted ossification. The condylar head also exhibited a porous trabecular architecture. Comparable structural changes were evident in the temporal fossa, supporting the concept that myeloid‐specific A20 deficiency affects craniofacial skeletal integrity beyond the dentoalveolar region.

**FIGURE 8:**
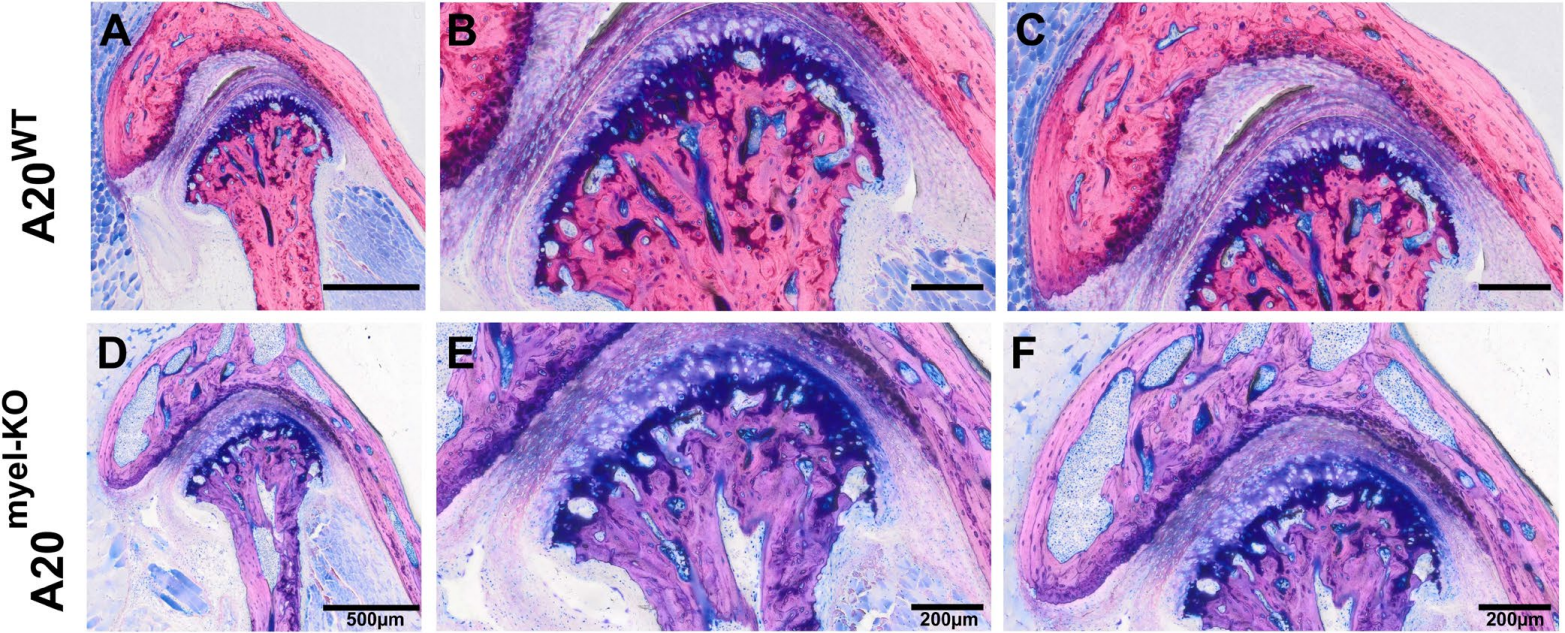
Temporomandibular joint phenotype by histology: Qualitative analysis. Histological photomicrographs of the TMJ in A20^*WT*^ (A–C) and A20^*myel‐KO*^ (D–F) mice. In A20^*WT*^ specimens, high‐magnification images show an intact articular cartilage surface with a well‐organized cartilage layer (B) and a compact, dense bone structure in the temporal fossa (C). In contrast, A20^*myel‐KO*^ exhibits alterations in condylar cartilage organization, including expansion of the proliferative zone and irregular hypertrophic zones, accompanied by a more porous trabecular bone structure in the condyle and temporal fossa.

## 4 Discussion

Periodontal tissue homeostasis is maintained through tightly regulated immune surveillance at the interface between host tissues and the oral microbiota [1, 2, 3]. Neutrophils, monocytes, and macrophages continuously respond to microbial and mechanical stimuli, coupling host defense mechanisms with physiological bone remodeling. These responses involve inflammatory signaling pathways such as NF‐κB, which coordinate antimicrobial activity but must be precisely controlled to prevent excessive tissue destruction [3]. In the present study, we investigated whether disruption of myeloid immune regulation through deletion of the NF‐κB inhibitor A20 affects craniofacial skeletal structures under baseline conditions [22]. Our findings revealed structural alterations in the dentoalveolar complex and temporomandibular joint of A20^*myel‐KO*^ mice, characterized by reduced alveolar bone volume fraction, widening of the periodontal ligament space, and compromised TMJ bone integrity.

Myeloid‐specific deletion of A20 produced a structural phenotype within periodontal supporting tissues, manifested by reduced alveolar bone volume and widening of the periodontal ligament space. These changes are consistent with altered bone remodeling and resemble phenotypes described in other skeletal compartments of myeloid A20‐deficient mice [17]. Because A20 functions as a negative regulator of NF‐κB signaling, these findings are compatible with the concept that dysregulated inflammatory signaling may influence bone homeostasis. However, the present study was designed primarily as a morphological characterization and did not directly assess molecular signaling pathways or inflammatory mediators. Therefore, the mechanisms linking A20 deletion to the observed skeletal alterations remain to be elucidated in future studies.

Interpretation of CEJ–ABC distances in this model requires consideration of additional structural parameters identified by micro‐CT. A20^*myel‐KO*^ mice exhibited reduced tooth volume and widening of the periodontal ligament space, which alters the spatial relationship between the cemento*–*enamel junction and the alveolar bone crest. Consequently, shorter CEJ–ABC distances in A20^*myel‐KO*^ specimens do not necessarily indicate less periodontal bone loss but rather reflect altered dentoalveolar morphology associated with changes in tooth dimensions and periodontal ligament architecture.

The TMJ represents a distinct craniofacial skeletal compartment that undergoes continuous cartilage and bone remodeling. Although the biological context of the TMJ differs from that of periodontal tissues, both structures are influenced by immune‐regulated bone turnover [17, 20]. The inclusion of TMJ analysis in this study was intended to determine whether the structural phenotype associated with myeloid A20 deletion extends beyond the dentoalveolar complex to other craniofacial skeletal sites. The observed reduction in bone volume fraction and alterations in cartilage organization in A20^*myel‐KO*^ mice suggest that dysregulated myeloid immune signaling may influence multiple craniofacial structures undergoing continuous remodeling. These findings are also consistent with previous observations that myeloid A20 deficiency is associated with inflammatory skeletal phenotypes beyond the oral cavity [17, 20].

In addition to mineralized tissues, our soft tissue analyses indicate that myeloid immune dysregulation may also be associated with changes in epithelial organization. In the oral lining mucosa, A20^*myel‐KO*^ showed a thinner epithelium with less pronounced rete ridges, suggesting altered epithelial–mesenchymal interactions and reduced epithelial complexity. Cytokines produced by myeloid cells, such as TNF‐α and IL‐1β, are known to influence epithelial proliferation and differentiation and may represent potential pathways contributing to the epithelial alterations observed [23, 24]. The junctional epithelium retained its characteristic non‐keratinized architecture in both groups, although cementum appeared thicker at the cemento*–*enamel junction in A20^*myel‐KO*^. In the hard palate mucosa, overall epithelial architecture was preserved in both groups, with only subtle qualitative differences in the appearance of the keratinized layer. Taken together, these observations are consistent with a role for A20 in immune–epithelial as well as immune–skeletal crosstalk within the craniofacial environment [25, 26].

From a translational standpoint, these data imply that variation in intrinsic negative‐feedback control of myeloid NF‐κB signaling may modulate periodontal susceptibility. The epithelial and mineralized tissue alterations in A20^*myel‐KO*^ are consistent with structural changes reported in chronic low‐grade periodontal inflammation and support the concept that host regulatory capacity influences the threshold for tissue breakdown. Interindividual differences in A20 expression or function could therefore contribute to heterogeneous clinical trajectories, including rapid progression despite limited plaque accumulation [15, 27]. In this framework, excessive myeloid NF‐κB activity may manifest as exaggerated inflammatory reactivity, impaired mucosal repair, and disproportionate remodeling responses to otherwise physiological stimuli. At the same time, any therapeutic implications of the A20–NF‐κB axis remain hypothetical in the context of the present morphological study and will require direct mechanistic and interventional investigation.

Several limitations should be acknowledged. Sample size was modest; however, bilateral craniofacial sites were analyzed, increasing the robustness of the morphological assessment. The study was performed under baseline conditions and did not test responses to defined periodontal challenges (e.g., ligature placement, pathogen exposure, or altered mechanical loading). Finally, we did not perform molecular profiling of effector pathways in stromal, osteoclast‐lineage, or cartilage‐associated cells. Such analyses will be essential to determine whether altered myeloid NF‐κB dynamics directly contribute to the downstream remodeling changes observed here.

## 5 Conclusions

In conclusion, this study provides a morphological characterization of craniofacial skeletal alterations associated with myeloid‐specific deletion of A20. Even without overt experimental challenge, loss of this checkpoint was associated with alveolar bone loss, periodontal ligament space expansion, epithelial changes, and TMJ structural deterioration. These results provide a structural framework for future studies investigating how the A20–NF‐κB regulatory axis may influence periodontal and craniofacial tissue remodeling.

## Disclosure

This manuscript used NLP (ChatGPT) for proofreading an entirely human‐generated text, but no other use of AI was made.

## Data Availability

The data that support the findings of this study are available on request from the corresponding author. The data are not publicly available due to privacy or ethical restrictions.
